# Supplementary material for: The Systems Biology Research Tool: evolvable open-source software
Source: BMC Syst Biol. 2008 Jun 29;2:55. doi: 10.1186/1752-0509-2-55 (PMC2446383; doi:10.1186/1752-0509-2-55)
Supplement: Additional file 1 — SBRT Archive. An archive of the current version of the Systems Biology Research Tool. [file 1752-0509-2-55-S1.zip › sbrt-1.4.0/doc/developers_guide/api/sbrt/kernel/math/geom/HashConvexPolytopeOld.html]

HashConvexPolytopeOld


|  |  |  |
| --- | --- | --- |
| **Overview** \| **Package** \| **Class** \| **Use** \| **Tree** \| **Deprecated** \| **Index** \| **Help** | |  |
| **PREV CLASS**   **NEXT CLASS** | **FRAMES**    **NO FRAMES**     **All Classes** |  |
| SUMMARY: NESTED \| FIELD \| CONSTR \| METHOD | DETAIL: FIELD \| CONSTR \| METHOD |  |


---


## sbrt.kernel.math.geom Class HashConvexPolytopeOld<V>

```
java.lang.Object
  sbrt.kernel.math.geom.HashConvexPolytopeOld<V>
```

**Type Parameters:**: `V` - the variable type.

**All Implemented Interfaces:**: ConvexPolytope<V>

---

``` public class HashConvexPolytopeOld<V> extends java.lang.Object implements ConvexPolytope<V> ```

This class is a concrete implemenation of `ConvexPolytope`.

**Author:**
:   This class was written and documented by
    Jeremiah Wright while in the Wagner lab.

---

**Constructor Summary**

`HashConvexPolytopeOld(LinearSystemSolution<V> systemSolution,
java.util.Map<? extends V,Interval> variableBounds,
double tolerance)`
  
          Constructs a new convex poltyope from the provided linear system
solution and variable bounds.


| **Method Summary** | |
| --- | --- |
| `int` | `dimensions()`             Returns the dimensions of the space in which this polytope lies. |
| `java.util.Set<ParallelHyperplanes<V>>` | `getBoundingPlanes()`             Returns the set of bounding hyperplanes that define this convex polytope. |
| `LinearSystemSolution<V>` | `getSystemSolution()`             Returns the linear system solution used to form this convex polytope. |
| `java.util.Set<V>` | `getVariables()`             Returns the variables contained in the hyperplanes defining the bounding half-spaces. |
| `boolean` | `isVariable(java.lang.Object variable)`             Indicates if the provided object is a variable in the bounding hyperplanes of this convex polytope. |
| `boolean` | `isWithin(java.util.Map<? extends V,java.lang.Double> point, double tolerance)`             Indicates if the provided point lies within this convex polytope. |

**Methods inherited from class java.lang.Object**

`clone, equals, finalize, getClass, hashCode, notify, notifyAll, toString, wait, wait, wait`

**Constructor Detail**

### HashConvexPolytopeOld

```
public HashConvexPolytopeOld(LinearSystemSolution<V> systemSolution,
                             java.util.Map<? extends V,Interval> variableBounds,
                             double tolerance)
```

:   Constructs a new convex poltyope from the provided linear system
    solution and variable bounds.

    **Parameters:**: `systemSolution` - a solution to a system of linear equations.: `variableBounds` - the bounds on each variable in that solution.: `tolerance` - the tolerance used to simplify the provided linear system solution. See `LinearSystemSolution.simplify(Map, double)`.


**Method Detail**

### getSystemSolution

```
public LinearSystemSolution<V> getSystemSolution()
```

:   Returns the linear system solution used to form this convex polytope.

    :   **Returns:**: the linear system solution used to form this convex polytope.

---


### getVariables

```
public java.util.Set<V> getVariables()
```

:   Returns the variables contained in the hyperplanes defining the bounding
    half-spaces.

    :   **Specified by:**: `getVariables` in interface `ConvexPolytope<V>`
    :   **Returns:**: the variables contained in the hyperplanes defining the bounding half-spaces.

---


### isVariable

```
public boolean isVariable(java.lang.Object variable)
```

:   Indicates if the provided object is a variable in the bounding
    hyperplanes of this convex polytope.

    :   **Specified by:**: `isVariable` in interface `ConvexPolytope<V>`
    :   **Parameters:**: `variable` - the potential variable. **Returns:**: `true` if the provided object is a variable in the bounding hyperplanes of this convex polytope; `false` otherwise.

---


### getBoundingPlanes

```
public java.util.Set<ParallelHyperplanes<V>> getBoundingPlanes()
```

:   Returns the set of bounding hyperplanes that define this convex polytope.

    :   **Specified by:**: `getBoundingPlanes` in interface `ConvexPolytope<V>`
    :   **Returns:**: the set of bounding hyperplanes that define this convex polytope.

---


### dimensions

```
public int dimensions()
```

:   Returns the dimensions of the space in which this polytope lies.

    :   **Specified by:**: `dimensions` in interface `ConvexPolytope<V>`
    :   **Returns:**: the dimensions of the space in which this polytope lies.

---


### isWithin

```
public boolean isWithin(java.util.Map<? extends V,java.lang.Double> point,
                        double tolerance)
```

:   Indicates if the provided point lies within this convex polytope.

    :   **Specified by:**: `isWithin` in interface `ConvexPolytope<V>`
    :   **Parameters:**: `point` - variables mapped to numerical values. Every key of this map must be a variable of this polytope and every variable must be present.: `tolerance` - the amount by which a point may lay outside this polytope, but still be considered within it. This should be a small positive numer, like 1E-9 for example. **Returns:**: `true` if the provided point lies within this polytope; `false` otherwise.


---


|  |  |  |
| --- | --- | --- |
| **Overview** \| **Package** \| **Class** \| **Use** \| **Tree** \| **Deprecated** \| **Index** \| **Help** | |  |
| **PREV CLASS**   **NEXT CLASS** | **FRAMES**    **NO FRAMES**     **All Classes** |  |
| SUMMARY: NESTED \| FIELD \| CONSTR \| METHOD | DETAIL: FIELD \| CONSTR \| METHOD |  |


---
